# HRP2 and HRP3 cross-reactivity and implications for HRP2-based RDT use in regions with *Plasmodium falciparum hrp2* gene deletions

**DOI:** 10.1186/s12936-021-03739-6

**Published:** 2021-04-29

**Authors:** Amy Kong, Scott A. Wilson, Yong Ah, Douglas Nace, Eric Rogier, Michael Aidoo

**Affiliations:** 1 Malaria Branch, Centers for Disease Control and Prevention, 1600 Clifton Road, Atlanta, GA 30329 USA; 2 The CDC Foundation, 600 Peachtree Street NE, Suite 1000, Atlanta, GA 30308 USA

**Keywords:** Malaria, RDT, HRP2, HRP3, Cross-reactivity, Gene deletions

## Abstract

**Background:**

The *Plasmodium falciparum* antigen histidine rich protein 2 (HRP2) is a preferred target for malaria rapid diagnostic tests (RDTs) because of its abundant production by the parasite and thermal stability. As a result, a majority of RDTs procured globally target this antigen. However, previous reports from South America and recent reports from sub-Saharan Africa and Asia indicate that certain *P. falciparum* parasites have deletions of the gene coding for HRP2. The HRP2 antigen is paralogous to another *P. falciparum* antigen HRP3 and some antibodies to HRP2 cross-react with HRP3. Multiple parasites have been described with deletions of one or both *hrp2* and *hrp3* genes. It is unclear how the various combinations of *hrp2* and *hrp3* deletion genotypes affect clinical sensitivity of HRP2-based RDTs.

**Methods:**

Cross-reactivity between HRP2 and HRP3 was tested on malaria RDTs using culture-adapted *P. falciparum* parasites with both *hrp2* and *hrp3* intact or with one or both genes deleted. Ten-fold serial dilutions of four culture-adapted *P. falciparum* parasites [3D7 (*hrp2*+/*hrp3*+), Dd2 (*hrp2*−/*hrp3*+), HB3 (*hrp2*+/*hrp3*−) and 3BD5 (*hrp2*−/*hrp3*−)] ranging from 100,000 to 0.01 parasites/µL were prepared. HRP2, *Plasmodium* lactate dehydrogenase (pLDH) and aldolase concentrations were determined for the diluted samples using a multiplex bead assay. The samples were subsequently tested on three RDT products designed to detect *P. falciparum* by HRP2 alone or in combination with pLDH.

**Results:**

At parasite densities of approximately 1000 parasites/µL, parasites that expressed either *hrp2* or *hrp3* were detected by all three RDTs. Multiplex based antigen measurement using HRP2- conjugated beads demonstrated higher antigen concentration when both *hrp2* and *hrp3* genes were intact (3D7 parasites, 47.9 ng/ml) compared to HB3 (3.02 ng/mL) and Dd2 (0.20 ng/mL) strains that had one gene deleted. 3D7 at 10 parasites/µL (0.45 ng/mL) was reactive on all three RDT products whereas none of the other parasites were reactive at that density.

**Conclusions:**

Above a certain antigen threshold, HRP3 cross-reactivity on HRP2-based RDTs is sufficient to mask the effects of deletions of *hrp2* only. Studies of *hrp2* deletion and its effects on HRP2-based RDTs must be studied alongside *hrp3* deletions and include clinical sample reactivity on HRP2-based tests.

## Background

Malaria diagnosis with rapid diagnostic tests (RDTs) has increased dramatically since the 2010 World Health Organization (WHO) recommendation that all suspected malaria cases be confirmed with a parasite-based test [1]. Globally, over 340 million malaria RDTs were sold by manufacturers in 2019 [2]. Due to the high prevalence of *Plasmodium falciparum* in sub-Saharan Africa, a majority of RDTs used in this region target that parasite, with most detecting the *P. falciparum-*specific antigen histidine rich protein 2 (HRP2). HRP2 is abundantly produced by the parasite [3, 4] and is highly stable within and outside the host, including retention in previously infected RBCs [5, 6]. Therefore, RDTs targeting HRP2 are more sensitive than RDTs targeting lactate dehydrogenase (pLDH) or aldolase, the other parasite antigens used in malaria RDTs [7]. As a result, and in accordance with global malaria guidance, most countries in sub-Saharan Africa with predominantly *P. falciparum* malaria prioritize HRP2-targeting RDTs either as a single antigen test or in combination with other antigens. Consequently, HRP2-targeting RDTs account for most of all global malaria RDT procurements [8].

Previous reports from South America, Asia, and recent reports from sub-Saharan Africa [9–14] suggest there are *P. falciparum* parasites with deletions of either or both genes coding for HRP2 and the related protein HRP3, rendering these parasites incapable of producing these antigens. Parasites with deletions of both these genes produce false negative results on HRP2-based RDTs and are therefore a threat to the use of HRP2-based RDTs [9, 14]. The genes coding for HRP2 and HRP3 are located on different chromosomes in the *P. falciparum* genome [15]. However, due to conserved repeating epitopes between both antigens, some monoclonal antibodies to HRP2 cross-react with HRP3 [16]. Consequently, HRP2-based RDTs can produce positive results when tested with samples from infections with parasites with *hrp2* deletions, but intact *hrp3* [17–19]. *Plasmodium falciparum* parasites have been identified with deletions in both *hrp2* and *hrp3* or singly deleted for one of these genes. This cross-reactivity has been reported using a highly sensitive HRP2-based RDT [17] and models using *hrp2*−/*hrp3*+ data from Kenya predict high density infections with such parasites could produce positive results on conventional RDTs [18]. However, better understanding of this interaction is needed for conventional RDTs that are more commonly used and expected to play a role in *hrp2* deletion surveillance. In addition, analyses of how HRP2 and HRP3 interact to produce a test result on HRP2-based RDTs is necessary to better design studies that determine the effects of *hrp2* deletions on the effectiveness of HRP2-based RDTs. In this study, the reactivity of serial dilutions of four culture-adapted *P. falciparum* strains with intact *hrp2* and *hrp3* or with one or both genes deleted on three different RDT products was determined. Extensive HRP3 cross-reactivity on HRP2 detecting RDTs is described.

## Methods

### Parasites

The following culture-adapted *P. falciparum* parasites were used: 3BD5 (*hrp2*−/*hrp3*−), Dd2 (*hrp2−*/*hrp3*+), HB3 (*hrp2*+/*hrp3*−), and 3D7 (*hrp2*+/*hrp3*+). *P. falciparum* 3BD5 was provided by the National Institute of Allergy and Infectious Diseases, National Institutes of Health under a material transfer agreement with CDC and was genotyped to confirm *hrp2* and *hrp3* deletion. The other parasites were available in the CDC Malaria Branch culture adapted parasite repository. Prior to their use in this study, HB3, Dd2 and 3D7 were genotyped using methods describe elsewhere [10, 19, 20] to confirm presence or absence of *hrp2* and *hrp3*.

The parasite strains were grown in culture using standard *P. falciparum* culture techniques [21]. All parasites were synchronized to the ring developmental stage using sorbitol. After satisfactory growth, parasite preparations were harvested and parasitaemia calculated by smear microscopy. Each preparation was diluted with PCR-confirmed malaria negative human group O+ blood to a parasitaemia of 100,000 parasites/µL after which serial tenfold dilutions were made to a low parasitaemia threshold of 0.01 parasites/µL. The malaria negative blood was from a malaria naïve individual residing in the US and procured from a blood bank. For each of the four parasites, multiple 60 µL aliquots of each dilution were prepared and frozen at −80 °C until used. Each aliquot was thawed once, and any remainder discarded after use.

In addition to parasitaemia, concentrations of HRP2, pLDH and aldolase were determined for each parasite dilution using a bead-based multiplex assay as previously described [22]. Serially diluted parasite samples were further diluted 1:20 for testing on the bead assay. Because the bead assay contained beads coupled to detection monoclonal antibodies raised against HRP2 and not HRP3, any HRP2 reactivity observed for the parasite Dd2 (*hrp2*−/*hrp3*+) was considered a cross-reactive detection of the same epitopes on HRP3. In addition, HRP2 reactivity detected for 3D7 (*hrp2*+/*hrp3*+) was considered a combination of detection of both HRP2 and cross-reactive HRP3.

### Rapid diagnostic tests

Three different RDT brands were used in the study. Two products, CareStart™ Malaria Pf (HRP2) Ag RDT and CareStart™ Pf/Pv (HRP2/pLDH) were from the same manufacturer; (AccessBio Inc, Somerset, New Jersey, USA) and the other, CareUs™ Malaria Combo Pf/PAN (HRP2/pLDH) Ag from a different manufacturer (CareUS, Seoul, South Korea). Antigen specificity for the tests were HRP2 (*P. falciparum* only), HRP2/Pv-LDH (*P. falciparum*/*P. vivax*) and HRP2/Pan-LDH (*P. falciparum*/pan-*Plasmodium*). All three RDTs met WHO recommended procurement criteria as determined by the WHO RDT product evaluation [23].

### RDT testing

RDTs were tested following manufacturer instructions. The only deviation was the use of micropipettes to transfer samples onto test cassettes instead of specimen transfer devices provided in the kits. The 10 dilutions for each parasite isolate were tested on the three RDT brands at the same time. Therefore, there were four testing sessions, one session for each parasite isolate.

RDT results were recorded in a MS Excel template. The template had fields for the following: time buffer was added to the test device and time test was read as well as presence and intensity of “control” and “test” bands. Bands were recorded as “0” for negative (no reactivity) or the numbers “4”, “3” “2” and “1” for positive tests with decreasing band intensities respectively. A band intensity color chart was used as a guide to determine band intensity as described in the WHO Malaria RDT product evaluation report [23].

### Analyses

RDT results were recorded in a results template developed in MS excel that included entries for band intensities for both control and test bands for each individual test. Each test was performed only once. For antigen concentrations in the bead assay, median fluorescence intensities (MFI) of sample wells minus background (MFI-bg) were compared to a standard curve derived from titration of recombinant antigens for HRP2, pLDH and aldolase as previously described [22]. Parasite density threshold for symptomatic malaria was estimated to be approximately 1000 parasites/µL based on previous reports [24].

## Results

### Parasites

**Fig. 1:**
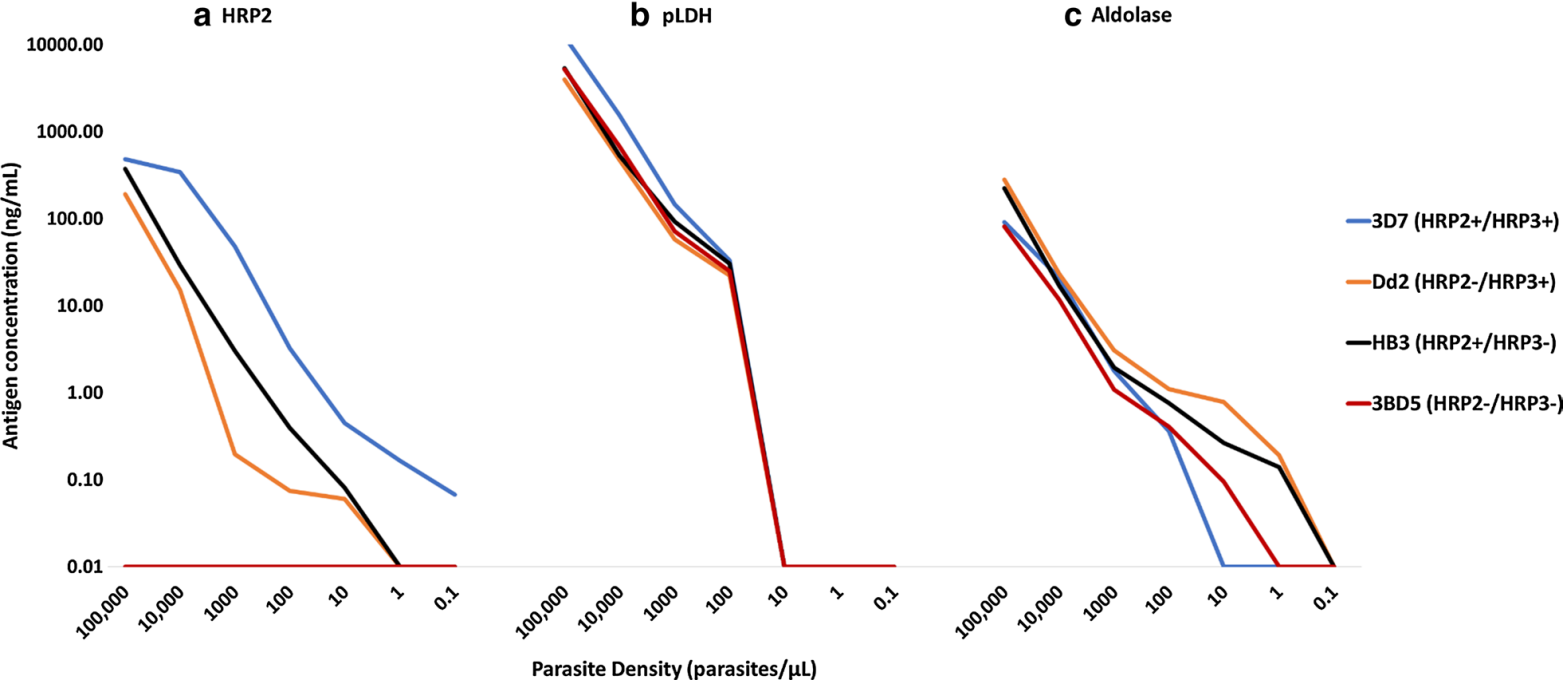
Concentrations of **a** HRP2, **b** p-LDH and **c** Aldolase detected by multiplex bead assay plotted against serial dilutions of 3D7, Dd2, HB3 and 3BD5. No HRP2 was detected for 3BD5 irrespective of parasite density. Note: y-axis is a log scale and antigen concentrations of 0.00 ng/mL are indicated on the chart as 0.01 ng/mL to allow for plotting

The culture-adapted parasites were successfully grown with parasitaemia ranging between 1% and 4.0% infected RBCs. The HRP2, pLDH and aldolase concentrations for each dilution of the four parasites were determined by multiplex bead assays (Fig. 1 and Table 1). All four parasites expressed both pLDH and aldolase and, as expected, had higher antigen concentrations associated with higher parasite densities. However, for 3BD5, no HRP2 was detected irrespective of parasite density, confirming deletion of both *hrp2* and *hrp3*. Concentrations of pLDH and aldolase were for the most part similar among the four parasite strains at the same parasite densities. However, HRP2 assay signal (and extrapolated concentration) was dependent on the *hrp2* and *hrp3* genotype. 3D7, capable of producing both HRP2 and HRP3, had the highest measured HRP2 concentration per parasite biomass. At the parasite density of 1000 parasites/µL, HRP2 concentration for 3D7 was 47.9 ng/mL compared to 3.02 ng/mL, 0.20 ng/mL and 0.0 ng/mL for HB3, Dd2 and 3BD5 respectively (Fig. 1 and Table 1).

**Table 1:** Antigen concentrations for culture parasite dilutions

Parasite density (p/µL)	3D7 (HRP2+/HRP3+)	Dd2 (HRP2−/HRP3+)	HB3 (HRP2+/HRP3−)	3BD5 (HRP2−/HRP3−)
	Antigen concentration (ng/mL)			
HRP2				
100,000	481.26	192.46	376.15	0.00
10,000	344.98	15.25	29.27	0.00
1000	47.86	0.20	3.02	0.00
100	3.25	0.08	0.40	0.00
10	0.45	0.06	0.08	0.00
1	0.17	0.00	0.00	0.00
0.1	0.07	0.00	0.00	0.00
pLDH				
100,000	12406.91	4007.06	5379.97	5213.57
10,000	1557.34	474.12	534.33	677.61
1000	146.28	57.74	92.89	72.14
100	33.06	22.19	30.46	24.83
10	0.00	0.00	0.00	0.00
1	0.00	0.00	0.00	0.00
0.1	0.00	0.00	0.00	0.00
Aldolase				
100,000	90.76	280.24	223.95	81.21
10,000	20.94	23.35	16.96	11.68
1000	1.78	3.06	1.94	1.09
100	0.36	1.11	0.76	0.41
10	0.00	0.78	0.27	0.10
1	0.00	0.19	0.14	0.00
0.1	0.00	0.00	0.00	0.00

### Cross-reactivity on HRP2 bands

**Fig. 2:**
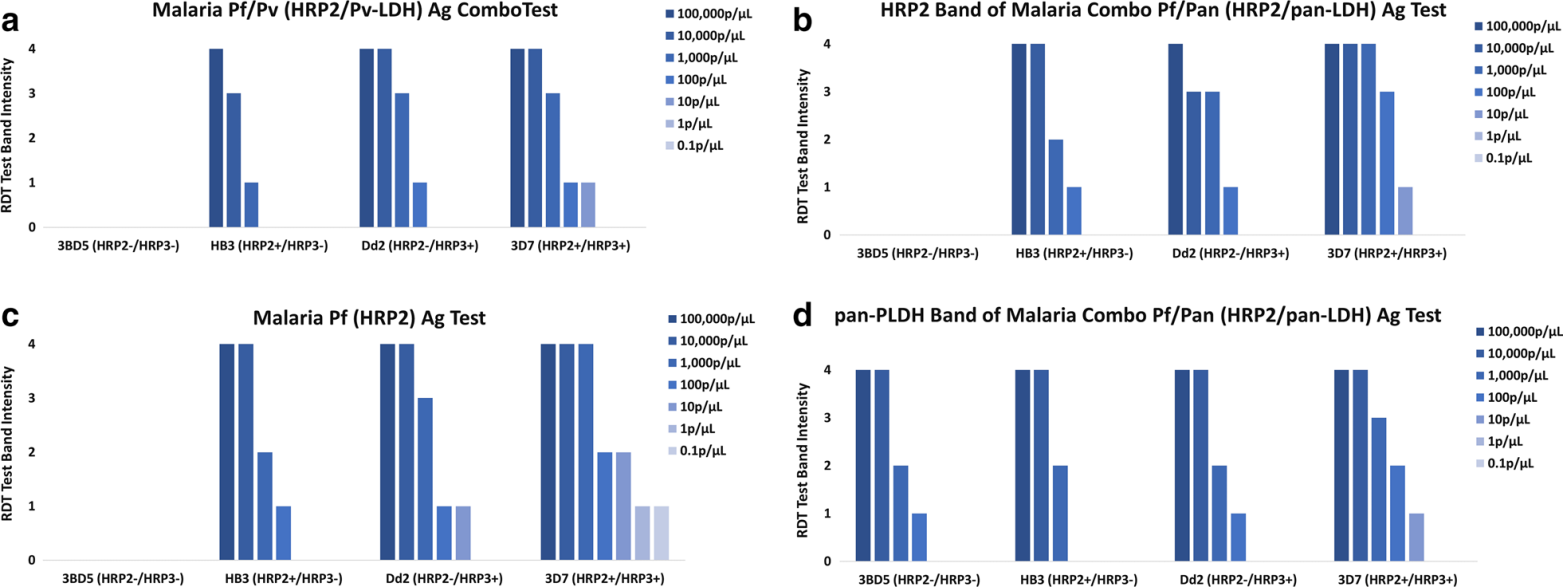
Reactivity of serially diluted culture parasites on RDTs showing relative band intensities. Band intensities are graded as 0 (negative) or 1–4 (positive with increasing band intensity respectively). Dd2 (*hrp2*− /*hrp3*+), was reactive on all HRP2 bands at 100 parasites/µL (**a**, **b**, **c**) and in one case at 10 parasites/µL (**c**). Only for the double deleted 3BD5 (*hrp2*− /*hrp3*−) was HRP2 reactivity completely lost irrespective of parasite density (**a**, **b**, **c**). All parasites were reactive on the pan-LDH band

At parasite densities of  ≥ 1000 parasites/µL [24], the *hrp2−*/*hrp3*+ parasite Dd2 was always detected on the HRP2 bands on all three RDTs used in this study. Measured antigen concentrations for other Dd2 parasite dilutions were 192.5 ng/mL, 15.3 ng/mL and 0.20 ng/mL for 100,000, 10,000 and 1000 parasites/µL respectively. Even with Dd2 at 100 parasites/µL (0.08 ng/ml antigen detected by HRP2-conjugated beads), a density often associated with asymptomatic infection in high transmission settings, HRP2 bands on all three RDTs were positive, although at low band intensities (Fig. 2). In addition, for one of the RDT products (HRP2 only), Dd2 at 10 parasites/µL (HRP2-bead assay determined concentration of 0.06 ng/mL) was positive with a band intensity score of 1 for the HRP2 band (Fig. 2c). Like Dd2, HB3 (*hrp2*+/*hrp3−*) was reactive on HRP2 bands on all RDT brands at parasite densities of  ≥ 1000 parasites/µL (HRP2 antigen concentration of 3.02 ng/mL at 1000 parasites/µL). However, reactivity at 100 parasites/µL (HRP2 antigen concentration of 0.40 ng/mL) was observed on only two of the three products tested (Fig. 2).

### Complete absence of HRP2 reactivity

Total loss of HRP2 band reactivity for all three RDT products was observed only for the *hrp2-*/*hrp3-* parasite 3BD5 (Fig. 2a–c). This observation was irrespective of parasite density. In addition, HRP2 antigen was not detected in any dilution of this isolate in the bead assay. In contrast to the HRP2 reactivity for this parasite, reactivity was observed on the pan-pLDH band of the one product targeting this antigen at parasite densities of 100,000, 10,000, 1,000 and 100 parasites/µL (Fig. 2d) with pLDH concentrations of 5213, 677, 72 and 25 ng/mL respectively (Fig. 1 and Table 1).

### Full complement HRP2 reactivity

HRP2 band reactivity was detected for the 3D7 isolate (*hrp2*+/*hrp3*+) at parasite densities of 100,000 to 10 parasites/µL (HRP2 antigen concentration range 481.3–0.45 ng/mL) for all RDT products and as low as 0.01 parasites/µL (HRP2 antigen concentration of 0.07 ng/mL) for the HRP2 only RDT (Fig. 2a–c). 3D7 also had higher measured HRP2 concentrations per parasite biomass than the other three isolates (Fig. 1).

### RDT pLDH band reactivity

All the parasite strains were reactive on the pan-LDH test at the parasite density of 1000 parasites/µL with a limit of detection of 10 parasites/µL for 3D7, 100 parasites/µL for 3BD5 and Dd2 and 1000 parasites/µL for HB3. Corresponding pLDH antigen concentrations as measured in the bead assay are shown in Table 1. As expected, none of the parasites irrespective of antigen concentration produced a positive test on the Pv-LDH bands of the HRP2/Pv-LDH test.

## Discussion

The presence in endemic regions of *P. falciparum* parasites lacking either or both *hrp2* and *hrp3* is recognized as a threat to malaria case management especially in sub-Saharan Africa, where the WHO recommends the use of HRP2 only RDTs. Exceptions to this recommendation are Ethiopia and Madagascar where *P. vivax* transmission occurs. The extent of these deletions is unclear because of inadequate surveillance. The WHO recommends a method of surveillance [25] that uses results from a combination of HRP2-based RDT with a pan-LDH RDT, Pf-LDH RDT or smear microscopy on clinical malaria cases with HRP2 false negative RDT result as an initial trigger for widespread *hrp2/hrp3* deletion surveillance. This surveillance protocol depends on phenotypic expression of HRP2 as the most important initial trigger rather than an initial investigation into costly molecular testing for *hrp2/hrp3* deletions. Because HRP2-specific antibodies can cross-react with HRP3, malaria patient sample reactivity on HRP2-based RDTs would be expected to be influenced by HRP3 when the gene is present in the infecting parasite. Understanding how this cross-reactivity influences HRP2 RDT reactivity is critical for conducting *hrp2*/*hrp3* deletion surveys and how policy changes away from HRP2-based RDTs are made.

Results presented here show that at parasite densities of  ≥ 1000 parasites/µL, often associated with symptomatic malaria infection in sub Saharan-Africa, *P. falciparum* parasites with either *hrp2* only or *hrp3* only deletions may not be distinguishable phenotypically from parasites with both genes intact when tested on HRP2 RDTs and that HRP2 RDTs remain useful except when both *hrp2* and *hrp3* are deleted. Indeed, for RDTs used in this study, cross-reactivity is seen even at lower parasite densities typically associated with asymptomatic infections in sub-Saharan Africa. These data imply that genotyping for *hrp2* only in a parasite population is insufficient to provide information needed to advocate for changes in testing policy in favor of non-HRP2-based tests. Complete loss of HRP2 reactivity at all parasite densities was only observed for the *hrp2*/*hrp3* double-deleted parasite 3BD5. An important observation that also needs to be considered for *hrp2*/*hrp3* deletion surveillance and which is emphasized by the WHO *hrp2*/hrp*3* deletion surveillance protocol [25] is that samples used for deletion surveillance by necessity should be high density infections. At 100 parasites/µL, parasites with *hrp2* or *hrp3* only deletions (Dd2 and HB3) showed similar reactivity patterns on HRP2 test bands. However, RDTs have decreased sensitivity at low densities and false negative RDT tests observed at such densities may reflect the lowered test sensitivity rather than a deletion of the relevant genes.

*Plasmodium falciparum* 3D7 with both *hrp2* and *hrp3* intact was observed on the bead assay to have a higher measured HRP2 concentration per parasite biomass at ≥ 1000 parasites/µL than the other parasites with single gene deletions. This observation is likely due to a combination of the parasite strain producing both HRP2 and the cross-reacting HRP3 as well as an inherent ability to produce more of the protein. In addition, HB3 (*hrp2*+/*hrp3*−) had higher HRP2 concentration than Dd2 (*hrp2*−/*hrp3*+) per parasite biomass due to the greater number of antibody epitopes on HRP2 or greater avidity for the homologous antigen compared to HRP3 [26]. While the distinction in HRP2 concentration of each parasite strain and dilution was clear on the quantitative bead assay, assay saturation point on the qualitative RDT means that any differences in HRP2 concentration between *hrp2* only or *hrp3* only parasites is lost at high (≥ 1000 parasites/µL) parasite densities. Therefore, when surveying for *hrp2*/*hrp3* deletions, false negative HRP2 RDT reactivity at higher parasite densities is likely to be more informative than false negative reactivity at low (~ 100 parasites/µL) densities. The cross-reactive pattern described here also means single *hrp2* or *hrp3* deletions are likely underestimated in parasite populations since these parasites, especially in high density clinical cases, will produce positive HRP2 RDT reactions and are, therefore, unlikely to be selected for genotyping. The pattern of pLDH reactivity largely reflected expectations with all parasites producing parasite density and antigen concentration-dependent reactivity on the pan-pLDH RDT and no reactivity on the Pv-LDH RDT.

A limitation of this study is that RDTs from only two manufacturers were used and products from other manufacturers could produce different results due to the use of different HRP2 antibodies. It remains to be tested how product-specific antibody types influence reactivity. However, this potential cross-reactivity requires that *hrp2* deletion surveys are not conducted in isolation but always in conjunction with *hrp3* deletion with the knowledge that cross-reactivity is extensive enough to mask the effects of *hrp2* only deletions and that HRP2 production phenotype as measured by RDTs is influenced by both genes. In addition, this potential cross-reactivity needs to be understood and addressed for any RDT being used as part of *hrp2*/*hrp3* deletion surveillance. An HRP3-specific antibody with no HRP2 cross-reactivity in the bead assay could also help with better resolution of cross-reactivity between the two antigens. Another limitation of the study is that, parasites with different genetic backgrounds have inherent differences in antigen production capabilities and although higher HRP2 antigen levels were measured for 3D7 compared to the same parasite concentration of the other parasites, the differences may be in part due to that parasite’s ability to produce more HRP2 and not necessarily only due to *hrp2* and *hrp3* being intact. Also, at the lower parasite densities, RDT reactivity did not always reflect the presence of antigen as measured in the bead assay, demonstrating the lower sensitivity of RDTs closer to their limits of detection. However, except for HRP2 for the 3BD5 parasite, there was a high positive correlation between antigen concentration and parasite density for all parasite strains (*r* range 0.83–0.99).

## Conclusion

Data presented here suggest a high level of cross-reactivity of HRP3 on routinely used HRP2-based RDTs. Studies of *hrp2* deletions and their possible effects on HRP2 RDTs should consider the presence or absence of *hrp3* and the potential reactivity of HRP3 on RDTs used in deletion surveillance.

## Data Availability

All data from this study are available upon reasonable request.

## Abbreviations

HRP2: Histidine-rich protein 2 (antigen)
*hrp2*: Histidine rich protein 2 (gene)
HRP3: Histidine-rich protein 3 (antigen)
*hrp3*: Histidine rich protein 3(gene)
LDH: Lactate dehydrogenase
RDT: Rapid diagnostic test
WHO: World Health Organization
